# Facilitating access to pneumococcal vaccine for people living with HIV: an experience report

**DOI:** 10.1590/1980-220X-REEUSP-2021-0563en

**Published:** 2023-01-06

**Authors:** Patricia da Silva Spindola Parmejani, Camila de Melo Picone, Ana Paula Pereira da Silva Alves, Ana Marli Christovam Sartori, Karim Yaqub Ibrahim

**Affiliations:** 1 Universidade de São Paulo, Faculdade de Medicina, Hospital das Clínicas, São Paulo, SP, Brazil.

**Keywords:** Immunizatio, Pneumococcal Vaccine, Vaccination Coverag, Acquired Immunodeficiency Syndrom, HI, Nursin, Inmunización, Vacunas Neumococicas, Cobertura vacunal, Síndrome de Inmunodeficiencia Adquirida, VIH, Enfermería, Imunização, Vacinas Pneumocócicas, Cobertura Vacinal, Síndrome da Imunodeficiência Adquirida, HIV, Enfermagem

## Abstract

The article describes a strategy to facilitate access to pneumococcal conjugate vaccine 13 (PCV-13) for people living with HIV/AIDS (PLHIV) during the COVID-19 pandemic.

**Method:**

report on the experience regarding the organization of a care service for PLHIV in the city of São Paulo to facilitate access to PCV-13 in the framework of the 2020 influenza vaccination campaign during the COVID-19 pandemic.

**Results:**

through the integration between a PLHIV care service and an Immunization Center (CRIE in Portuguese), it was possible to offer PCV-13 to PLHIV at the point of care, reducing physical barriers to access to immunization. Thus, of the 1,906 PLHIV who passed through the service during the period March 23-July 31, 2020, 84.4% (1,609) received the influenza vaccine, PCV-13 or both. Of the 1609 vaccinated, 50.6% (814) were eligible and received PCV-13.

**Conclusion:**

offering the vaccine at the point of care and orienting PLHIV on the importance of vaccination as a disease prevention strategy, identifying those eligible to receive it, was an important action carried out by the institution together with the nursing team, as a strategy to facilitate access to vaccination.

## RESUMO

Descrever uma estratégia para facilitar o acesso à vacina conjugada pneumocócica 13-valente (PCV-13) para pessoas vivendo com HIV (PVHIV), durante a pandemia de COVID-19.

**Método:**

relato de experiência sobre a organização de um serviço de atendimento para PVHIV na cidade de São Paulo, para facilitar o acesso à PCV-13 no decorrer da campanha de vacinação de influenza de 2020, durante a pandemia de COVID-19.

**Resultados:**

por meio da integração entre um serviço de atendimento para PVHIV e um Centro de Imunizações (CRIE) foi possível oferecer a PCV-13 para as PVHIV em seu local de atendimento, diminuindo barreiras físicas de acesso à imunização. Dessa forma, das 1906 PVHIV que passaram pelo serviço durante o período de 23 de março a 31 de julho de 2020, 84,4% (1609) receberam a vacina influenza, PCV-13 ou ambas. Dos 1609 vacinados, 50,6% (814) foram elegíveis e receberam a PCV-13.

**Conclusão:**

oferecer a vacina em seu local de tratamento e orientar as PVHIV sobre a importância da vacinação como estratégia de prevenção de doenças, identificando os elegíveis a recebê-las, foi uma importante ação realizada pela instituição em conjunto com a equipe de enfermagem, como estratégia de facilitar o acesso à vacinação.

## RESUMEN

El artículo describe una estrategia para facilitar el acceso a la vacuna neumocócica conjugada 13 (PCV-13) a las personas que viven con VIH/SIDA (PVVS) durante la pandemia de COVID-19.

**Método:**

relato de experiencia sobre la organización de un servicio de atención a las PVVS en la ciudad de São Paulo, para facilitar el acceso a la PCV-13 en el marco de la campaña de vacunación contra la gripe de 2020, durante la pandemia de COVID-19.

**Resultados:**

a través de la integración entre un servicio de atención a las PVVS y un Centro de Inmunización (CRIE), fue posible ofrecer la PCV-13 a las PVVS en su punto de atención, reduciendo las barreras físicas para el acceso a la inmunización. Así, de las 1.906 PVVS que pasaron por el servicio durante el periodo comprendido entre el 23 de marzo y el 31 de julio de 2020, el 84,4% (1.609) recibieron la vacuna de la gripe, la PCV-13 o ambas. De los 1609 vacunados, el 50,6% (814) eran elegibles y recibieron la PCV-13.

**Conclusión:**

ofrecer la vacuna en el lugar de atención y orientar a las PVVS sobre la importancia de la vacunación como estrategia de prevención de enfermedades, identificando a las personas elegibles para recibirlas, fue una acción importante realizada por la institución junto con el equipo de enfermería, como estrategia para facilitar el acceso a la vacunación.

## INTRODUCTION

Even though highly effective antiretroviral therapy (ART) has reduced the incidence of pneumococcal disease (PD) among people living with HIV/AIDS (PLHIV), Streptococcus pneumoniae (SP) is still a common cause of pneumonia and invasive disease (meningitis and sepsis) in this population^(1,2)^.

Many factors may influence PD morbidity and mortality in PLHIV, including regional causes such as pathogen prevalence, access to adequate treatment and ART, and host-related aspects such as advanced age, presence of comorbidities, smoking, recreational drug use, and degree of HIV-related immunosuppression^(1,2)^.

In addition, PD is a major public health problem worldwide because of the volume of hospitalizations, costs, and resistance of SP to antibiotics^(3,4,5)^. Vaccination represents the best preventive strategy for pneumococcal disease, especially among the most vulnerable (PLHIV, cancer patients, and individuals undergoing bone marrow and solid organ transplantation).

Since the 1980s, the United States Centers for Disease Control has recommended the use of the 23-valent polysaccharide pneumococcal vaccine (PPV-23) for immunocompromised individuals^(6)^.

However, as a polysaccharide vaccine, PPV-23 induces T-cell-independent immune response and therefore does not induce memory B cells, which reduces the duration of vaccine protection, thus making revaccination necessary, even in the immunocompetent host^(7,8)^.

It has also been reported that repeated booster doses of PPV-23 may cause “hypo-responsiveness.” This limitation makes PPV-23 less suitable for PLHIV, especially those in advanced stages of immunodeficiency^(8)^.

Pneumococcal conjugate vaccines (PCV), on the other hand, induce T-cell-dependent immune responses with long- lived memory cells, prevent B-cell depletion and result in the production of high-affinity antibodies. These can also prepare the immune system for more rapid and enhanced responses to booster doses^(7,8)^.

The 13-valent pneumococcal conjugate vaccine (PCV-13) includes purified capsular polysaccharides from 13 SP serotypes (1, 3, 4, 5, 6A, 6B, 7F, 9V, 14, 19A, 19F, 18C, and 23F) and was licensed by the U.S. Food and Drug Administration (FDA) in 2010^(9)^.

Immunogenicity data suggest that PCV-13 induces similar immune response (for some serotypes) to PPV-23 in adults, including PLHIV^(10)^. Based on this information, PCV-13 may be a good basic PD prophylaxis strategy for PLHIV.

Within the context of disease prophylaxis, in Brazil, the National Immunization Program (PNI in Portuguese) of the Ministry of Health (MoH), formulated in 1973, is responsible for the control, elimination and/or eradication of immune- preventable diseases through vaccination, in addition to ensuring the quality and diversity of immunobiological products offered to the population. This program, is both nationally and internationally recognized for its actions in this scenario, also performing the logistics of distribution of immunobiological products through the network and cold chain throughout the country^(11)^. It is worth mentioning that with the creation of the Unified Health System (SUS in Portuguese) in 1988, the access to the various health services became universal for the entire population of the country^(12)^, ensuring greater equity of PNI’s actions and expanding the number of immunobiological products available to meet various segments of the population, such as the immunosuppressed^(11)^. These immunobiological products, considered special, became available in strategic places with easy access to this population, such as the Reference Centers for Special Immunobiological products (CRIE in Portuguese)^(13)^.

The CRIE are services administratively linked to the institution where they are located (hospitals, outpatient clinics), and technically to the State Health Secretariats. They provide personalized services to the population in need of high technology and high-cost products. Through the CRIEs, the PNI articulates with other departments of the MoH, such as the Chronic Conditions Diseases and Sexually Transmitted Infections, to include new technologies in its program and offer them to the population in need, including those living with HIV^(13)^.

Thus, PCV-13 was incorporated into the PNI and made available at the CRIEs by the end of 2019. This vaccine is now offered to people over 5 years old with HIV, cancer patients, individuals undergoing hematopoietic stem cell transplantation, and solid organ transplant recipients^(14)^.

The PNI advises to follow a sequential schedule with PCV-13 and PPV-23, i.e., a PCV-13 dose followed by a PPV-23 dose two months later. For people who have already received PPV-23, a one-year interval is recommended before administering PCV-13^(14)^.

In this context, PCV-13 vaccination is indicated for all PLHIV in Brazil, regardless of TCD4+ lymphocyte count or HIV viral load; and expanding access to this preventive measure to combat PD is an important public health strategy.

However, this public health strategy has been weakened with the risk of a pandemic scenario. At the end of 2019, the World Health Organization (WHO) was notified regarding an outbreak of pneumonia, in Wuhan city, Hubei province, People’s Republic of China^(15)^. Subsequently, a new type of coronavirus (SARS-COV-2) was identified as the causer of this outbreak. In the early months of 2020, the world was already facing a Public Health Emergency of International Concern (PHEIC) for the newly identified disease named as “COVID-19”^(16)^.

Upon the arrival of this new disease in Brazil, individual and collective sanitary measures were instituted by official agencies to try to contain its advance, according to the different epidemiological scenarios, as well as the organization of health services to assist this demand^(17)^.

The physical isolation and quarantine measures instituted to reduce the transmission of the COVID-19 led to a reduction in the demand for health services and in particular for routine vaccination, resulting in a decrease in vaccination coverage worldwide and also in Brazil^(18)^.

Faced with this new pandemic scenario added with a reduced flow of people who travel to seek health resources in different locations, health services, especially those serving PLHIV needed to create strategies to optimize care. Among these strategies include the realization of integration between services to make new health resources available in their units, such as the PCV-13 vaccine recently made available by the PNI to this population.

Some studies report that, aside from the fact that vaccination coverage in PLHIV is low for some vaccines, seeking it elsewhere constitute an important barrier to access vaccination^(19,20)^, with a clear increase in adherence when this service is available in the same place of care^(21)^.

Therefore, the objective of this report is to describe the strategy adopted by a reference service for the care of PLHIV to facilitate access to the 13-valent pneumococcal conjugate vaccine (PCV-13) during the pandemic of COVID-19.

## METHODS

This is a retrospective, descriptive, experience-report study on a strategy to facilitate access to vaccination with PCV-13, during the National Influenza Vaccination Campaign, carried out in a Reference Center for care for PLHIV in the city of São Paulo, from March 23 to July 31, 2020, during the pandemic of COVID-19.

The Outreach Service for People Living with HIV (Serviço de Extensão ao Atendimento de Pessoas Vivendo com HIV - SEAP in Portuguese) is a referral service for outpatient care for PLHIV, linked to a tertiary/quaternary university hospital, Clinical Teaching Hospital of the School of Medicine, University of Sao Paulo (HCFMUSP in Portuguese).

SEAP-HCFMUSP provides medical and interdisciplinary care for adults living with HIV, viral hepatitis (VH), and sexually transmitted infections (STI) in the western region of the city of São Paulo. In 2020, assistance was provided to 211 people living with viral hepatitis, 3,500 PLHIV, and 300 people on HIV pre-exposure prophylaxis (PrEP). The service operates Monday through Friday, and is located in Pinheiros, approximately 4 km from the CRIE, located in the HCFMUSP Outpatient Clinic Building.

Every year, the MoH organizes a national influenza vaccination campaign in the fall, between April and May. During these campaigns, the CRIE-HCFMUSP makes the influenza vaccine available to all healthcare professionals and target patients throughout the hospital complex, including the SEAP - HCFMUSP.

This report was built from institutional documents organized to facilitate the logistics of work between SEAP-HCFMUSP and CRIE-HCFMUSP (flows and operational procedures), in addition to the reports of professionals involved in this activity.

## ETHICAL ASPECTS

This report is compliant with the rules of Resolution 466/2012, of the National Health Council, and was approved by the Research Ethics Committee of the Department of Infectious and Parasitic Diseases of the Faculty of Medicine, University of São Paulo, under opinion number 001/2021.

## RESULTS

Having availability of PCV-13, and taking advantage of the logistical structure organized for the influenza vaccination campaign and the need to reduce the displacement of people due to the COVID-19 pandemic, the SEAP-HCFMUSP organized, together with the CRIE-HCFMUSP to break the physical barriers, vaccine logistics management and professional training, in order to offer the new vaccine for PLHIV, at its points of service, during the 2020 influenza vaccination campaign.

PLHIV that were being followed up in this service were considered eligible to receive a dose of PCV-13 when referred by a SEAP infectious disease physician or who presented a vaccination card proving that they had not received a dose of PCV-13 previously and/or VPP-23 in the last year, according to the standards recommended by the PNI/MoH.

It is noteworthy that for this activity, the trained nurses administered the vaccine not withstanding having or not a prescription, as long as they were eligible to receive it. For this to occur, all professionals of the nursing team were oriented and trained in the management of influenza and PCV-13 vaccination, according to the recommendations of the CRIE - HCFMUSP and PNI/MoH.

As a consequence of the COVID-19 pandemic, the SEAP-HCFMUSP carried out several strategies to reduce the flow of people in the institution, among these were the postponement of elective appointments, prioritizing face-to-face care only for complex cases, such as individuals with cardiovascular comorbidities, renal failure, or neoplasms that required clinical management. In addition, a nursing triage was implemented at the entrance of the service to detect suspected cases of COVID-19, following recommendations, among them that of the Federal Council of Nursing (COFEN in Portuguese)^(22)^.

Benefitting from the convenience of this setting, all people who arrived at the institution, and went through the nursing triage in SEAP, were informed about the annual influenza vaccination campaign and availability of PCV-13, in addition to receiving guidance on the importance of vaccination as a form of prevention of communicable diseases. PLHIV who were interested in receiving the vaccine and had no symptoms of COVID-19 were referred to the Nursing Sector, located on the 1st floor of the same service. After vaccination, everyone received guidance on care with the application site and possible local and/or systemic adverse events.

The information generated from the care for PLHIV, in the nursing triage, were stored in an Excel spreadsheet (Microsoft Excel) containing the variables such as age, sex, vaccines administered and whether the PCV-13 vaccine was sought, through prescription from the infectious disease physician of SEAP.

The records of the vaccine doses applied were forwarded by the nursing team at the end of the day for registration in the Immune System (vaccine registration system of CRIE-HCFMUSP).

A descriptive statistical analysis was performed using all records generated by the nursing triage and the applied doses of vaccines, in order to organize and present the results.

### Vaccine Distribution, Transportation, and Storage Logistics

The joint action of SEAP and CRIE, both from HCFMUSP, organized logistics for supplying influenza and PCV-13 vaccines.

The number of doses of each vaccine withdrawn by SEAP at CRIE was calculated based on the average number of weekly visits performed by SEAP. Thus, it was estimated a weekly average of 50 (fifty) doses of PCV-13 and 100 (one hundred) doses of influenza vaccine, which were withdrawn from the CRIE - HCFMUSP, at the beginning of each week, in the morning, packed in a sealed and identified thermal box appropriate for the transport of immunobiologicals, respecting the temperature between 2 and 8ºC, and transported to the SEAP - HCFMUSP, where the vaccines are stored in the pharmacy sector, in the chamber for immunobiologicals. The temperature of this chamber was checked three times a day by the nursing team and recorded in an institutional document.

The amount needed for daily use was transferred to an appropriate thermal box, with reusable coils, keeping the temperature between 2 and 8ºC. In order to maintain this temperature, the coils were changed when necessary.

To ensure the quality of storage and availability of vaccines to meet the needs of PLHIV, daily stock and temperature control documents were created. The temperature control was performed daily and in three distinct periods.

Owing to the fact that the service does not operate on weekends, and there is no generator to ensure proper temperature control in case of power failure, at the end of each week the surplus doses were properly transported back to the CRIE - HCFMUSP.

### Continuing Education of the Nursing Team

The SEAP nursing team consists of four nurses, four technicians, and three nursing assistants. To perform the daily vaccination activities, one nurse and two technicians or nursing assistants were scheduled per shift (4 hours/day schedule). This activity was developed from 10am to 6pm, with two periods set aside for changing shifts among the teams. There was also an administrative officer to perform the registration of vaccines in the system.

To meet the vaccination demand, the nursing team and the administrative officer were trained, according to the recommendations of the CRIE - HCFMUSP.

For the administrative officers, there was orientation and training on the use of the *Imuni System*, carried out by the Information Technology team of HCFMUSP.

In the first moment, the nurses were oriented by the CRIE-HCFMUSP team and, later, a manual on the management of vaccination for influenza and PCV-13 was made available.

Based on this manual, a lecture was structured for training the entire nursing team, in which the recommendations and benefits of vaccine protection for the target population, application method, dosage, and adverse events were presented. In order to ensure the continuity of these guidelines, and ensure patient safety during vaccination, nurses remained in attendance and supervision throughout the vaccination campaign.

### Population Vaccinated

**Figure 1.**
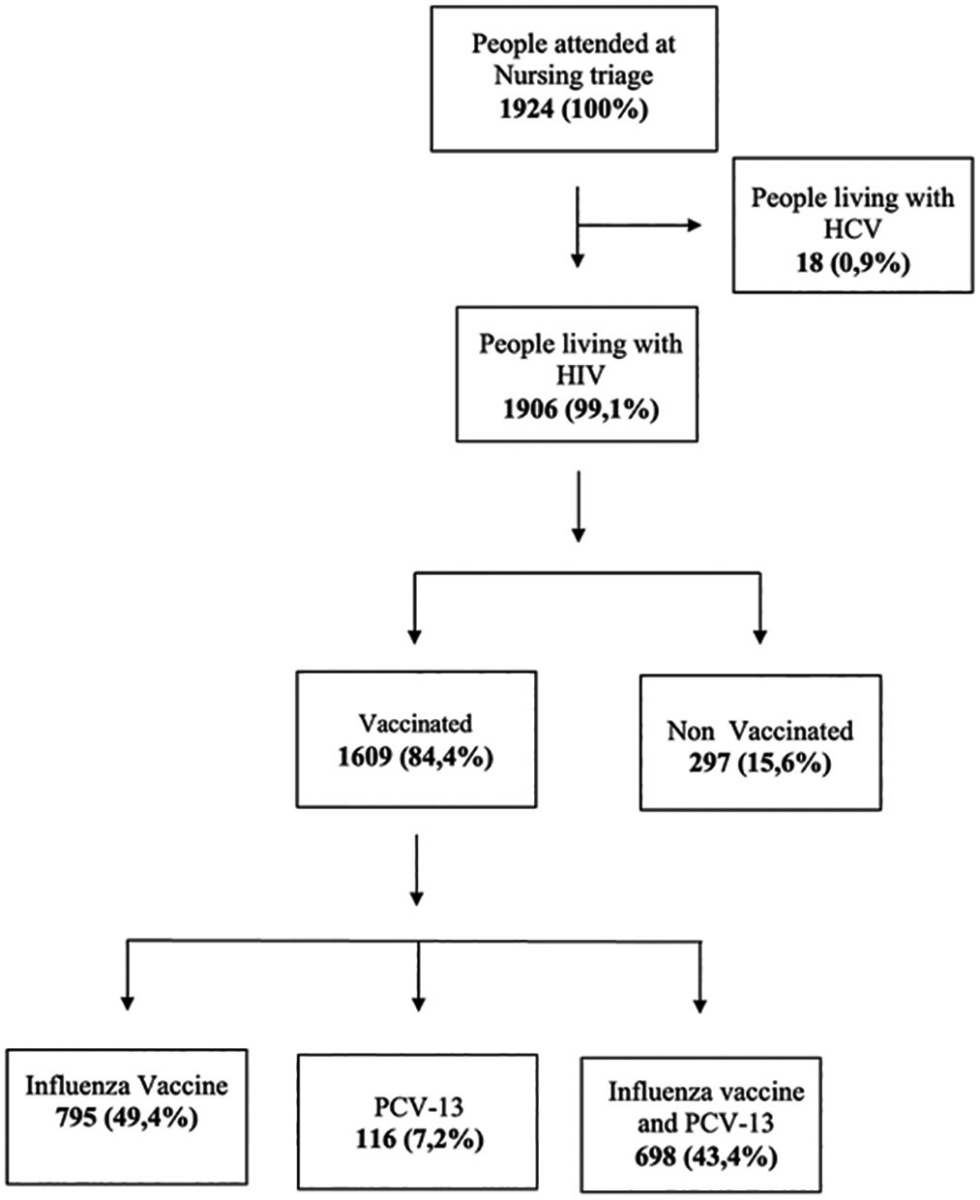
People seen at SEAP – HCFMUSP and vaccinated during the campaign, according to medical diagnosis and type of vaccine received. São Paulo, 2020.

During the period March 23 - July 31, 2020, the service recorded 3776 visits of 1924 people to the nursing triage sector. Of these, 1609 (83.8%) PLHIV were vaccinated while seeking other care at the service during the vaccination campaign period, according to Figure 1.

Among the services sought in the SEAP-HCFMUSP, only 454 (23.6%) people had medical consultation scheduled during the outpatient clinic, between 7 am and 7 pm, the others sought other care, such as psychology, social service, drug dispensing in the pharmacy, test collection, among others.

Among 814 PLHIV who received PCV-13, the majority (601) were referred for vaccination by nursing orientation, and the others were referred by prescription from the infectious disease specialist at SEAP. Seventy-one PLHIV received only the influenza vaccine on their first visit to the service, but later returned with their vaccination card to receive PCV-13.

The reasons for not administering PCV-13 to a PLHIV during the campaign were: having already received the vaccine, not presenting the vaccination card, or having received a dose of PPV-23 with an interval of less than a year.

It is noteworthy that among the 1924 people who went through the nursing screening, 1906 (99.1%) were PLHIV. This number represents 54.5% of the PLHIV population who were assisted by the institution.

## DISCUSSION

The present experience report exposed the articulation between the coordination of the institution together with the nursing team to perform the integration between services (SEAP and CRIE), finding a strategy to facilitate access to PCV-13 vaccine for PLHIV, in their place of care, thus managing to overcome physical barriers, logistics management of vaccines and professional training. This action is reflected in the number of people vaccinated at the institution, because during the pandemic, even with physical isolation, reduced patient flow and restricted vaccination hours, it was possible to facilitate access to PCV-13 for 814 (23.3%) among the 3,500 people in follow-up at SEAP, just by making it available to PLHIV at their point of care.

Enabling access to this new vaccine, besides being considered in this report as a way to offer a health product in a place where it was not performed before, also results from the training of nurses to evaluate the possibility of administering the vaccine without the need to require a medical prescription to perform PCV-13, since all patients were known to be HIV-positive, consequently, included in the recommendations of antipneumococcic vaccines by the PNI. It is worth remembering that, at national level, the PNI requires a prescription and medical report only for special immunobiologicals^(13)^.

It is noteworthy that, as in other Brazilian studies, the continuing education of nursing professionals assumed a prominent role so that they could be offered, with quality, information about the protection of vaccine-preventable diseases^(23,24)^. This action provided the dialogue with other health professionals of the institution to exchange knowledge and updates on the vaccination calendar and special immunobiologicals, as well as created the opportunity to perform health education with the assisted population during the nursing triage care and after vaccination. These spaces were essential to allow the construction of knowledge about this issue, and to help develop health autonomy and sharing of responsibility for care in this population.

Besides these practices of continuing education for the team and health education for the population, nurses, as well as the nursing team, play an important role in the organization, monitoring, administration, and conservation of immunobiologicals, in the disposal of waste resulting from the vaccination process, and in the control of immunopreventable diseases. Therefore, the nursing professional is an essential part for the operation of the national immunization program^(25,26)^.

In contrast to the Brazilian scenario, a study conducted by the International Council of Nurses with member countries of the Organization for Economic Cooperation and Development (OECD) showed barriers to the performance of nurses in their practices in immunization programs in their countries, such as the requirement of medical prescription for vaccination, work overload and lack of adequate training^(27)^.

On the other hand, some obstacles to access vaccination were also found. Among the PLHIV assisted by the nursing team in the period, 15.6% (297) did not receive any vaccination. Despite the performance of nursing in the enrollment and guidance of patients to vaccinate, there are some barriers that can be pointed out as the cause of non-vaccination of these people, such as the very reinforcement in medical guidance for vaccination, lack of motivation and understanding of the importance of vaccination demonstrated by the patient, having received PPV-23 in less than 12 months or not presenting the vaccination card.

There is a process that still needs to be improved regarding how to get PLHIV to be used to bring their vaccination card to medical appointments. Many people do not consider it a health document, resulting in missed opportunities for vaccination updates. As in Brazil, the PPV-23 is recommended for PLHIV for many years, it is necessary to evaluate this document so that there is no divergence in conduct in relation to the recommendations of the PNI^(13)^.

The computerization of health records is a valuable strategy to mitigate the impact of not using this document, avoiding missed opportunities for vaccination. This action has been occurring since the implementation of the Information System of the National Immunization Program (SIPNI in Portuguese), which allows the identification of the vaccinated individual and the risk assessment as to the occurrence of outbreaks and epidemics of immunopreventable diseases^(28)^, and advanced with the availability of the application “Conecte SUS”, created to allow access to users, to their health information in SUS, among these the digital vaccination certificate^(29)^.

Considering the perspective that this scenario benefits the information management, it is worth noting that this is not the reality of all health services. Although the SEAP- HCFMUSP is a computerized service, access to the system of consultation of vaccines performed was not available in this service, rendering impossible to use this tool.

However, even bearing in mind the difficulties triggered by the pandemic and some structural limitations, the services managed to integrate and organize a strategy that provided greater access to vaccination for PLHIV, which shows that integration between services can generate better vaccination coverage for this population.

This strategy can be expanded and maintained within the institution, and serve as well as a model for other services.

## CONCLUSIONS

The integration among different services can break down barriers of access to health actions. In the described strategy, it was possible to present a simple and effective measure to optimize PCV-13 vaccination to PLHIV at their point of care, improving the integration between a care service for PLHIV and a CRIE. This model also serves to optimize access to other vaccines recommended for this population. However, it should be noted the scarcity of studies in the literature discussing this topic.

In addition to increasing the knowledge regarding PLHIV about a new vaccination recommendation, this strategy at the point of care, also raises an alert to the physicians who are responsible for the recommendations and prescriptions of vaccines, raising awareness and motivating the multiprofessional team in respect to this issue.

It is important to highlight the fundamental role of the nursing team in organizing, together with the service coordination, all the logistics of implementation, team training and guidance to patients, raising the awareness in regards to vaccines in disease prevention.
